# The Influence of Interatrial Conduction Disorders on Atrial Mechanical Function — Atrial Strain and Pulmonary Veins Reversal Flow in Patients with COVID-19

**DOI:** 10.31083/RCM25273

**Published:** 2025-02-12

**Authors:** Jacek Zawadzki, Jacek Gajek, Grzegorz Zawadzki, Agnieszka Sławuta, Bartosz Kudliński

**Affiliations:** ^1^Department of Anesthesia, Critical Care and Rescue Medicine, Collegium Medicum, University of Zielona Góra, 65-729 Zielona Góra, Poland; ^2^Department of Social Sciences and Infectious Diseases, Wrocław University of Science and Technology, 58-376 Wrocław, Poland; ^3^Students Scientific Society, Department of Emergency Medical Service, Wrocław Medical University, 50-367 Wrocław, Poland; ^4^Department of Cardiology, Klodzko County Hospital, 57-300 Klodzko, Poland

**Keywords:** bachmann bundle, interatrial block, P-wave, pulmonary veins, atrial strain

## Abstract

**Background:**

The physiological activation of the left atrium (LA) happens through the Bachmann bundle, which is crucial for the heart's proper functioning. Bayes de Luna first described interatrial blocks (IABs) in 1979, noting their disruption of atrioventricular (AV) synchrony. This study aims to evaluate LA mechanics by analyzing LA strain in cases of normal and impaired interatrial conduction, focusing on retrograde flow in the pulmonary veins (PV).

**Methods:**

The study included 51 patients who tested positive for SARS-CoV-2 and exhibited related symptoms. Six patients with persistent atrial fibrillation (AF) were excluded from the study (45 patients qualified in total: 23 males, 22 females; mean age 69.0 ± 12.9 years).

**Results:**

IABs were more frequently observed in COVID-19 patients. Thus, they were included despite SARS-CoV-2 being a potential limitation of the study. All participants underwent clinical evaluation, electrocardiography (ECG) (200 mm/s ×256), and echocardiography to assess left ventricular ejection fraction (LVEF), mitral regurgitation (MR), LA volume, global and regional strain, and retrograde flow in the PV. A statistical dependency was found between LA global strain and P-wave morphology, MR, heart failure (HF), and paroxysmal atrial fibrillation (PAF). However, no clear correlation was found between retrograde flow in the PV and LA strain. The mean P-wave duration correlated with its morphology. Additionally, correlations were observed between P-wave morphology and hypertension, being overweight, and PAF.

**Conclusions:**

LA mechanics are negatively influenced by IABs. LA global strain correlates with P-wave duration, ejection fraction (EF), and MR independently. Regional LA strain examination is potentially effective for assessing LA mechanics and complements precise ECG.

## 1. Introduction

Sinus activation originates from the sinus node and spreads anteriorly to the right atrium (RA) and subsequently to the left atrium (LA) [1]. LA activation is mediated by the Bachmann bundle, a group of muscular fibers crucial for efficient interatrial conduction [2]. This efficient conduction is essential for the proper electromechanical activation of the LA, which ensures optimal filling of the left ventricle (LV) [3, 4]. Efficient conduction through the Bachmann bundle also contributes to the correct “valvular” function of the pulmonary vein (PV) outlets. Consequently, the circular fibers around the venous openings reduce their diameter, preventing blood from flowing back and protecting the lungs against blood retention [5]. Inter-atrial and atrioventricular (AV) conduction disturbances impair mechanical AV synchrony, which is more harmful in the left heart due to higher filling pressure [6]. Interestingly, the lengthening of both P-wave duration and AV conduction often occurs sequentially as a compensatory mechanism. Uncompensated pathologies resulting in abnormal AV synchrony and suboptimal LV filling may lead to heart failure (HF), particularly in the form of HF with preserved ejection fraction (HFpEF) [7]. A specific pathology is the complete block of the Bachmann bundle (advanced interatrial block (IAB)), which results in activation through alternative pathways [8]. In the classic form of advanced IAB, the duration of the P-wave is extended, accompanied by a typical change in morphology. Abnormal electrical activation is followed by abnormal mechanical LA contraction, which is logical. The wave moves from the base towards the openings of the superior PV, which significantly impairs LV filling and may contribute to the development of HF [9].

## 2. Purpose

This study aimed to evaluate the mechanical function of the LA using LA strain in patients with normal and impaired interatrial conduction, specifically focusing on contractility and retrograde flow in the upper and lower pulmonary veins. It also aimed to prove that the precise interpretation of electrocardiography (ECG) using vector graphics is complementary to LA strain assessment in terms of impaired function in IABs.

## 3. Material and Methods

The study included 51 unselected patients (25 women, 26 men) with a mean age of 69.5 ± 13.1 years. The patients were admitted to the University Hospital in Zielona Góra with a positive SARS-CoV-2 test and related symptoms. Inclusion criteria included sinus rhythm at the time of examination. IABs were more frequently observed in COVID-19 patients; hence, these patients were included despite SARS-CoV-2 potentially being a study limitation. Exclusion criteria included heart rhythm other than stable sinus rhythm at the time of examination, moderate and severe valvular heart disease, hemoglobin <11 mg/dL, estimated glomerular filtration rate <30 mL/min/1.73 m^2^, presence of malignancy, autoimmune disease, or thyroid illness. Six patients with persistent AF were excluded from the study -45 patients (23 males, 22 females; mean age 69.0 ± 12.9 years) eventually qualified for the study.

All participants underwent clinical evaluation, ECG, echocardiography, and blood sampling for laboratory analysis. Two independent researchers performed the examinations, unaware of each other’s results and blinded to clinical data. All study subjects were informed of the purpose and provided written informed consent. The study adhered to the Declaration of Helsinki and was approved by the local Bioethical Committee at Collegium Medicum University of Zielona Góra, Poland.

**Fig. 1.**
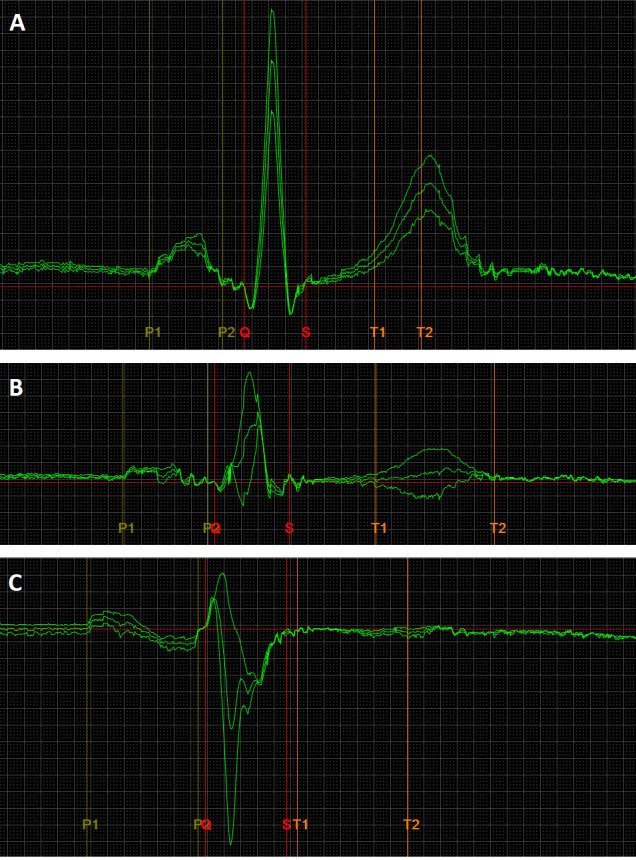
**An exemplary image of ECG recordings, presenting normal (A), long and flat – low voltage (B), full Bachmann bundle block (C) P-wave morphologies**. The ECG recording parameters were: speed 200 mm/s, gain 80 mm/mv. The ECG leads are II, III, aVF. The P-wave durations are as follows: (A) 110 ms, (B) 130 ms, (C) 166 ms. ECG, electrocardiography.

The ECG was interpreted using vector graphics at a recording speed of 200 mm/s with ×256 enhancement (Biomedical Instruments Co. Ltd, TeleECG-12C, Shenzen, China) (Fig. 1). Interatrial conduction disorders were defined based on the original definition published by Bayes de Luna in 1979 and further developed in 2017 [10, 11]. Advanced IAB diagnosis was performed by analyzing the 12-lead ECG. As presented in Fig. 1, patients were categorized based on P-wave morphology (especially in leads II, III, and aVF) and duration:

Group 1: Positive P-wave shape with a duration of up to 120 ms and amplitude above 0.1 mV in lead I [12].

Group 2: Partial interatrial block (P-IAB) with “long and flat” P-wave morphology characteristic of a structurally damaged atrium, with a duration exceeding 120 ms and an amplitude below 0.1 mV in lead I.

Group 3: Advanced interatrial block (A-IAB) with P-wave morphology described by Bayes de Luna as a positive and negative deflection of the P-wave (two phases) - “plus/minus” morphology and duration exceeding 120 ms [8].

Echocardiographic examination assessed ejection fraction (EF), mitral regurgitation (MR), LA peak global longitudinal strain in phases (reservoir [peak atrial longitudinal strain (PALS)], conduit, contraction [peak atrial contraction strain (PACS)]) using two-dimensional tissue tracking (2DTT) technology, LA regional strain (qualitative assessment focusing on the order of regional contractions), LA volume, and the pressure and speed of PV retrograde wave using FujiFilm Corporation Arietta 65. The volumes of the LV and LA were evaluated using the biplane Simpson and area-length methods, respectively. LV end-diastolic and end-systolic volumes in the apical 4- and 2-chamber views were used to calculate EF. The retrograde flow pressure in superior and inferior PV was measured with pulse wave Doppler, placing the marker approximately 1 cm inside the PV inlet. LA strains were assessed by semi-automated 2DTT speckle tracking technology by FujiFilm Corporation, analyzing all segments of LA in the apical 4- and 2-chamber views with a temporal resolution of 60 to 90 frames/s, using the onset of the QRS complex as the reference point. PALS was calculated as the peak value of longitudinal strain during LV systole. PACS was measured as the strain value at the onset of the P-wave in the ECG, and LA conduit strain was determined as the difference between PALS and PACS. The peaks of regional strain curves were set on a timeline, which helped define the LA regional contraction order. No separate quantification of regional strain values was used. The LV inflow parameters, including peak early (E) and late diastolic flow velocity (A), and deceleration time of the early diastolic flow wave (DT), were assessed from the apical 4-chamber view by pulsed-wave Doppler (Hitachi Ltd., Tokyo, Japan) with the sample volume.

## 4. Statistics

The collected data were registered, processed, and analyzed using Statistica 13.3 (TIBCO Software Inc., Palo Alto, CA, USA). Statistical analyses included qualitative variables measured on nominal and ordinal scales, which were cross-tabulated. The correlation strength between pairs of variables was assessed using the Chi-square test. Fisher’s exact test was used when the expected count in at least one cell of a four-field table was below 5. In cases of quantitative variables, means (M), standard deviations (SD), medians (Me), lower quartiles (Q1), upper quartiles (Q3), and ranges (minimum and maximum) were calculated. The Shapiro-Wilk test was used to assess the normality of distribution, while homogeneity of variance was assessed using Bartlett’s and Levene’s tests. Student’s *t*-test was used for the significance of differences between the mean values of variables with normal distribution and homogeneous variances in two independent groups. The non-parametric Mann-Whitney U test was used to verify the significance of differences between mean values of variables with non-normal distribution or heterogeneous variances in two groups. For multiple comparisons, analysis of variance (ANOVA) or its non-parametric equivalents were used.

Regression analysis, based on the Pearson r linear correlation coefficient, was applied to determine the direction and the strength of linear correlations between two continuous variables. The least square method was used to estimate regression coefficient values. Multiple regression analysis was conducted to assess the influence of different factors on the dependent variable. In multiple regression models, regression coefficients (β) were standardized to make their values independent of the value range of the associated random variable (standardized regression coefficients β range between –1 and +1, allowing comparison for different random variables; the higher the absolute value of the standardized regression coefficient, the stronger the variable’s impact on the dependent variable). A significance level of *p* = 0.05 was used for all statistical analyses. The results of the statistical analyses are included in graphs or tables.

## 5. Results

### 5.1 General Correlations

The clinical baseline characteristics are presented in Table 1. Table 2 displays the data excluding comorbidities and categorizes it into three groups based on P-wave morphology. The results of the significance and independence tests indicated no statistical relationship between P-wave morphology and gender, presence of MR, parameters of retrograde flow in the superior and inferior PV (measured in cm/s and mmHg), or the E/A ratio (*p* > 0.05). Conversely, age, P-wave duration, PALS, PACS, LA volume, and LVEF were found to correlate with P-wave morphology.

**Table 1.** **Characteristics of patients (COVID-19)**.

Variable		Statistics
Sex:		
	Male, *n* (%)	23 (51.0)
	Female, *n* (%)	22 (49.0)
P-wave duration (ms):		
	*Mean* ± *SD*	133.9 ± 17.4
	*Min*–*Max*	106–173
Age (years):		
	*Mean* ± *SD*	69.0 ± 12.9
	*Min*–*Max*	34–96
P-wave morphology:		
	Normal, *n* (%)	18 (40.0)
	Long and flat, *n* (%)	12 (26.7)
	Bachmann’s block, *n* (%)	15 (33.3)
Global Longitudinal Atrial Strain Reservoir (PALS) (%)		
	*Mean* ± *SD*	22.3 ± 10.0
	*Min*–*Max*	3.6–46.0
Global Longitudinal Atrial Strain Conduit (%)		
	*Mean* ± *SD*	11.2 ± 5.2
	*Min*–*Max*	1.7–24.6
Global Longitudinal Atrial Strain Contraction (PACS) (%)		
	*Mean* ± *SD*	12.5 ± 6.1
	*Min*–*Max*	1.7–25.5
EF (%)		
	*Mean* ± *SD*	43.3 ± 12.7
	*Min*–*Max*	17.9–65.7
Left atrial volume (mL)		
	*Me* [*Q*1–*Q*3]	32 [25–45]
	*Min*–*Max*	12–81
Mitral regurgitation (yes), n (%)		16 (31.4)
Superior PV retrograde flow speed *v* (cm/s)		
	*Mean* ± *SD*	27.6 ± 4.9
	*Min*–*Max*	18.1–38.2
Superior PV retrograde flow pressure *p* (mmHg)		
	*Me* [*Q*1–*Q*3]	0.3 [0.2–0.4]
	*Min*–*Max*	0.1–0.6
Inferior PV regurgitation speed *v* (cm/s)		
	*Mean* ± *SD*	28.2 ± 5.7
	*Min*–*Max*	15.9–44.0
Inferior PV regurgitation pressure *p* (mmHg)		
	*Me* [*Q*1–*Q*3]	0.3 [0.2–0.4]
	*Min*–*Max*	0.1–0.8
E/A index:		
	E/A <1, *n* (%)	23 (51.1)
	E/A >1, *n* (%)	22 (48.9)
	Hypertension, *n* (%)	36 (70.6)
	CKD, *n* (%)	2 (3.9)
	HF, *n* (%)	12 (23.5)
	IHD, *n* (%)	12 (23.5)
	Asthma, *n* (%)	3 (5.9)
	COPD, *n* (%)	7 (13.7)
	Obesity, *n* (%)	5 (9.8)
	DM2, *n* (%)	21 (41.2)
	AFP, *n* (%)	11 (21.6)

Characteristics of patients including variables such as sex, P-wave duration, age, P-wave morphology, global longitudinal atrial strain (reservoir, conduit, contraction), EF, LA volume, MR, PV retrograde flow speed and pressure, E/A index, hypertension, chronic kidney disease (CKD), heart failure (HF), ischemic heart disease (IHD), asthma, chronic obstructive pulmonary disease (COPD), obesity, diabetes mellitus type 2 (DM2), and paroxysmal atrial fibrillation (PAF); EF, ejection fraction; LA left atrium; MR, mitral regurgitation; PV, pulmonary veins; E/A, peak early/late diastolic flow velocity. n, number; (%), percentage; SD, standard deviation; Me, median; Q1, lower quartile; Q3, upper quartile; Min, minimum; Max, maximum.

**Table 2.** **Characteristics of patients in groups differing in the P-wave morphology and the results of tests of significance and independence**.

Variable	P-wave morphology			*p*-value
	Normal	Long and flat	Bachmann’s block	
	N = 18	N = 12	N = 15	
Male (yes)	9 (50.0)	8 (66.7)	6 (40.0)	0.590
P-wave duration (ms)	124.4 ± 15.2	138.5 ± 10.4*	141.7 ± 19.7*	0.007
Age (years)	63.8 ± 12.2	66.3 ± 14.2	73.6 ± 11.2*	0.034
PALS (%)	29.5 ± 6.0	20.0 ± 12.2*	19.6 ± 8.1*	<0.001
Global Longitudinal Atrial Strain Conduit (%)	12.5 ± 3.9	10.4 ± 7.2	10.3 ± 4.6	0.395
PACS (%)	16.9 ± 4.1	9.6 ± 6.0*	9.3 ± 5.1*	<0.001
LVEF (%)	52.9 ± 9.6	43.4 ± 10.4	38.2 ± 10.9	<0.001
LA volume (mL)	27 [19–31]	42 [34–45]	30 [21–45]	<0.001
Mitral regurgitation (yes)	5 (27.8)	7 (58.3)	2 (20.0)	0.090
Superior PV retrograde flow: *v* (cm/s)	26.8 ± 4.4	26.1 ± 4.6	29.4 ± 5.4	0.167
Superior PV retrograde flow: *p* (mmHg)	0.3 [0.2–0.4]	0.3 [0.2–0.4]	0.4 [0.2–0.4]	0.302
Inferior PV retrograde flow: *v* (cm/s)	29.6 ± 6.4	27.4 ± 4.8	26.8 ± 5.4	0.353
Inferior PV retrograde flow: *p* (mmHg)	0.4 [0.2–0.4]	0.3 [0.2–0.4]	0.3 [0.2–0.3]	0.382
E/A ratio <1 (yes)	7 (38.9)	7 (58.3)	9 (60.0)	0.406

*, The presence of statistical differences with normal group. PALS, peak atrial longitudinal strain; PACS, peak atrial contractile strain; LVEF, left ventricular ejection fraction; LA, left atrium; PV, pulmonary veins; E/A, peak early/late diastolic flow velocity.

### 5.2 Detailed Findings

⚫ P-wave Duration: Significant differences in P-wave duration were observed between normal conditions and P-IAB (*p* = 0.021) and between normal conditions and A-IAB (*p* = 0.003). No significant difference was found between P-IAB and A-IAB (*p* = 0.609). Thus, statistical differences in P-wave duration are present only between normal and abnormal P-wave morphologies.

⚫ Age: Significant age differences were found only between normal conditions and A-IAB (*p* = 0.026). No other significant differences were observed between other P-wave morphologies.

⚫ PALS and PACS: PALS and PACS showed similar trends (inversely) to P-wave duration. Statistical differences were noted only between normal conditions and P-IAB (PACS; PALS *p* = 0.002; *p* = 0.004) and between normal conditions and A-IAB (PACS *p* < 0.001; PALS *p* = 0.001). No statistical differences were present between the two abnormal P-wave morphologies (PACS *p* = 0.983; PALS *p* = 0.901), providing consistency between electrocardiographic and echocardiographic results.

⚫ LA Volume: The Kruskal-Wallis test did not find statistical differences in LA volume among the studied groups, indicating a need for further research on the origins of IABs.

⚫ Comorbidities: Hypertension (*p* = 0.023), obesity (*p* = 0.038), and paroxysmal atrial fibrillation (PAF) (*p* < 0.001) were statistically associated with P-wave morphology.

### 5.3 LA Global Reservoir Strain 

**Fig. 2.**
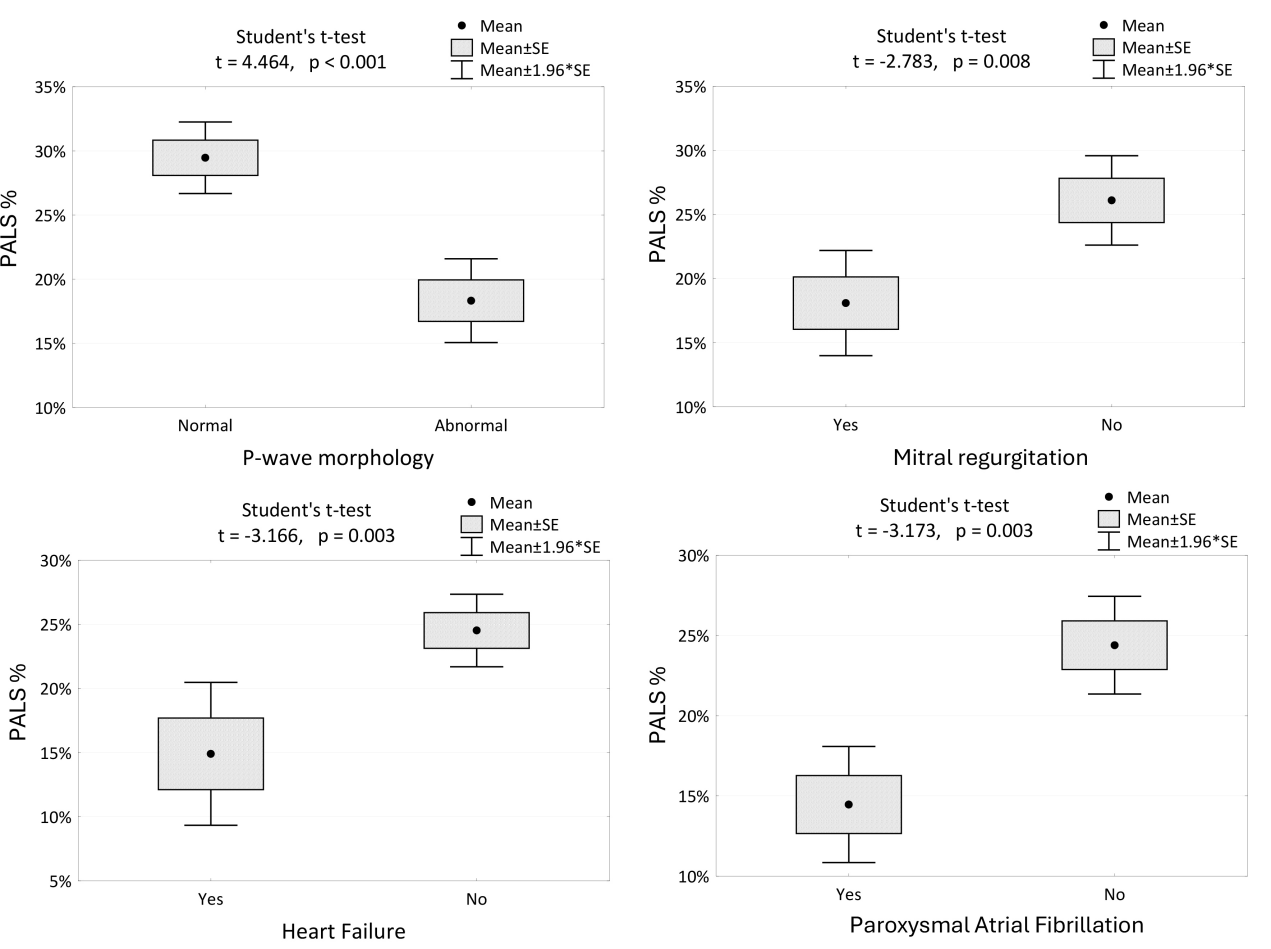
**The changes in PALS among the patients with different P-wave morphologies, the presence of mitral regurgitation, HF and AFP**. PALS, peak atrial longitudinal strain; PACS, peak atrial contraction strain; SE, standard error; HF, heart failure; AFP, paroxysmal atrial fibrillation.

Fig. 2 shows a statistical dependency between PALS and various parameters. P-wave morphology (normal/abnormal) and the presence of MR, HF, or AFP were significantly correlated with PALS.

Regression Models:

PALS positively correlates with LVEF and negatively correlates with P-wave duration. The model explains 47.5% of the variability in PALS.

PALS = 44.5 + 0.31 * LVEF – 0.262 * P-wave duration

R^2^ = 0.475

The model explains 47.5% of PALS variability.

PACS positively correlates with LVEF and negatively correlates with LA volume. The model explains 53.9% of the variability in PACS.

PACS = 7.7 + 0.25 * LVEF – 0.201 * LA Volume

R^2^ = 0.539

The model explains 53.9% of the variation in PACS.

### 5.4 LA Asynchrony and Regional Strain

**Fig. 3.**
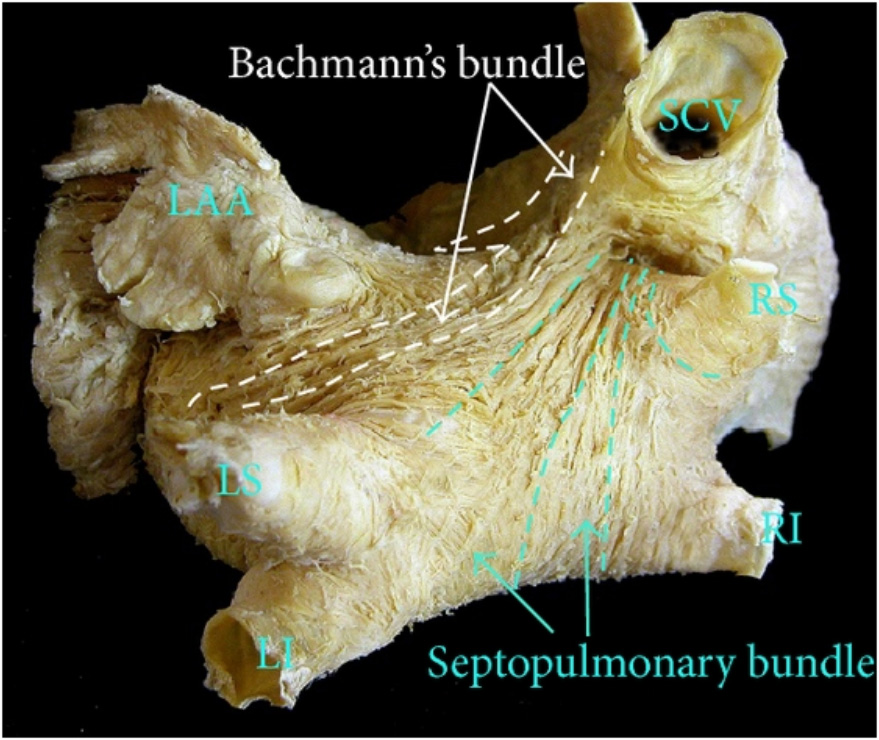
**The anatomic view of LA, with the approximate location of Bachmann and the septopulmonary bundle**. Sanchez-Quintana originally used the photo in his research. This photo is used as a courtesy of the author [13]. LAA, left atrial appendage; SCV, superior vena cava; LS, left superior pulmonary vein; LI, left inferior pulmonary vein; RS, right superior pulmonary vein; RI, right inferior pulmonary vein; LA, left atrium.

**Fig. 4.**
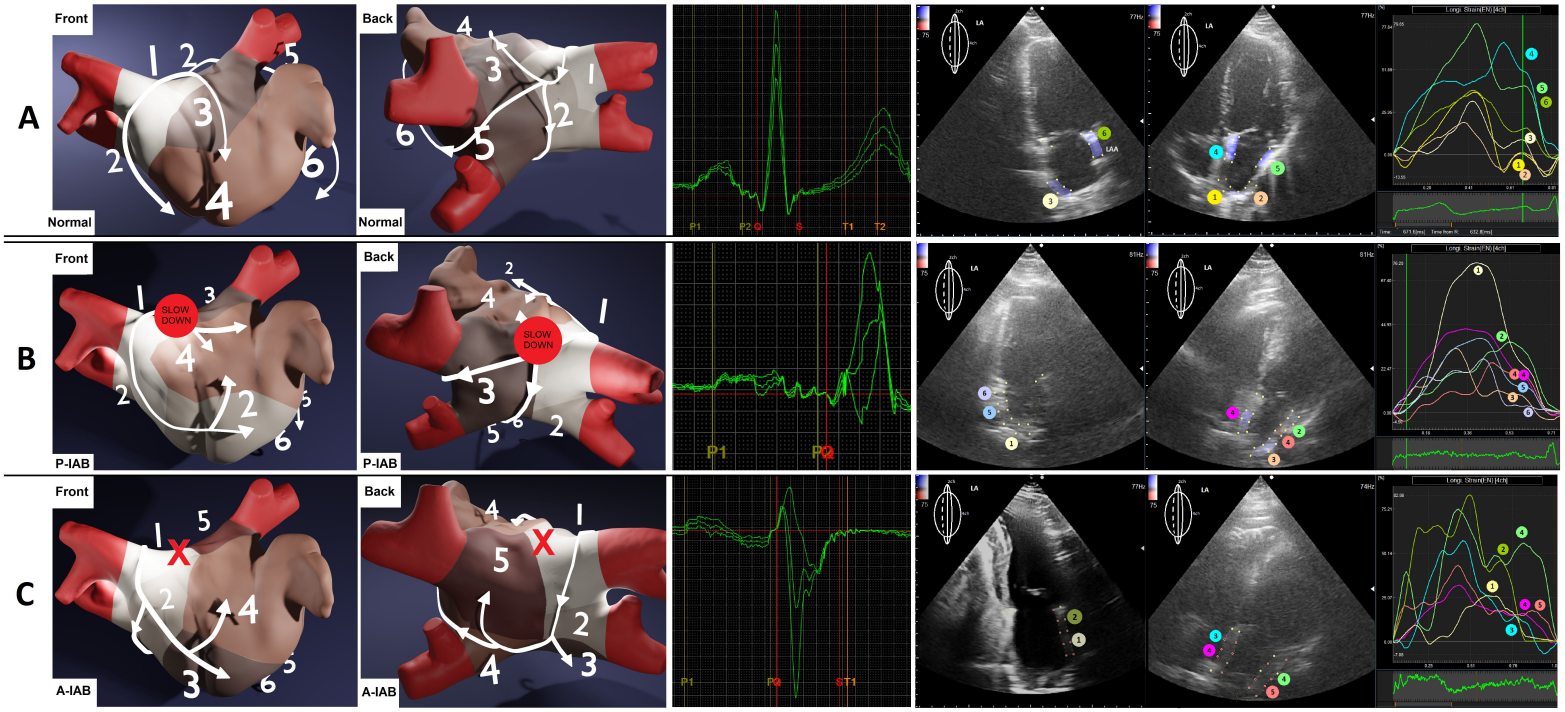
**The approximated differences in contraction profiles, based on analyzing LA regional strain, about P-wave morphologies**. Numbers and colors represent the order of contraction. (A) Normal P-wave. (B) Long’n’flat P-wave, P-IAB (the red spot). (C) Positive-negative P-wave, with A-IAB (the red X) — i.e., the conduction in the region on the Bachmann bundle is completely blocked. P-IAB, partial interatrial block; A-IAB, advanced interatrial block; P-IAB, partial interatrial block; A-IAB, advanced interatrial block; LA, left atrium; LAA, left atrial appendage.

Analyzing the peaks of regional strain curves revealed the contraction order of LA regions, which helped define the average direction of signal spread. We visualized the results in a 3D representation supported by an anatomical photo (Fig. 3 (Ref. [13]), Fig. 4).

### 5.5 Power of the Tests

Assuming a significance level of *p* = 0.05, the power of the Student’s *t*-test for the difference in mean global atrial strain parameters between the regular P-wave morphology group and the long and flat P-wave morphology group is 1–β = 0.862. The test power between the regular and Bachmann bundle block P-wave morphology groups is 1–β = 0.918. In both cases, the test power exceeds the minimum acceptable value of 0.8, which indicates sufficient patients in the subgroups.

## 6. Discussion 

Our primary achievement was demonstrating that the presence of IABs impair the synchrony of LA contraction, as evidenced by LA regional strain analysis. This method complements precise ECG assessment. Moreover, we identified factors directly influencing PALS and PACS.

### 6.1 Pathophysiology of IABs

Different conduction pathologies, as described by Bayes de Luna, provide insight into the mechanisms of IABs [8, 10, 11].

⚫ Full Conduction Block (A-IAB): This occurs when the roof of the LA or the junction between the LA and the right atrium (RA) is blocked. The exact location of the block is crucial because it determines whether the impulse has already entered the LA before the block or if it must use alternative pathways. The block is rarely located at the junction but instead on the roof of the LA [14, 15]. When the signal enters the LA but encounters the block, it travels anteriorly and inferiorly, surrounding the blocked region, resulting in caudocranial activation in the area of the PV. This phenomenon has been confirmed by Ramdat, who mapped the complete transversal conduction block of the Bachmann bundle (A-IAB) [16]. Our observations of similar contraction patterns based on regional LA strain indicate that LA regions contract in an order consistent with the spreading wavefront in the PV area, highlighting echocardiography as a less invasive alternative to epicardial mapping.

⚫ Partial Conduction Block (P-IAB): This involves slowed activation in the roof of the LA due to damaged conduction fibers, resulting in a prolonged, double-peaked P-wave on ECG [17]. The first peak reflects RA activation, while the second reflects LA activation. Occasionally, additional parallel activation through the foramen ovale or coronary sinus fibers may occur, leading to a long, flat, and irregular P-wave morphology [18]. Our observations indicate that this “long and flat” morphology in P-IAB occurs more frequently than the typical double-notched P-wave of advanced IAB. Platonov reported numerous P-wave morphologies [19], but the hemodynamic implications and prognostic values remain unclear.

Interestingly, alternative conduction pathways may exist in both A-IAB and P-IAB [20, 21]. Our results suggest a significant statistical dependency between normal conditions and any IAB but not between P-IAB and A-IAB, supporting the concept of a “grey zone” between these conditions.

### 6.2 Strain Technology and Measurements

Speckle tracking technology, which measures changes in the length of regions over time, underpins our strain analysis [22]. This technology has evolved significantly since 1991 [23, 24, 25]. In 2021, Kupczyńska *et al*.’s review [26] of LA strain knowledge established reference values and measurement techniques. Research has demonstrated correlations between LA strain measurements and LV diastolic dysfunction, aiding in early HFpEF diagnosis [27, 28]. This prompted the inclusion of LVEF in our study. Most research focuses on raw percentage values and their correlations with other parameters [29]. Watanabe, however, discussed LA mechanical dispersion in 2015, defined as the standard deviation of time to peak positive strain corrected by the R-R interval, a parameter reflecting atrial fibrosis and electrophysiological disorders [30]. This inspired our focus on LA regional strain curves to confirm LA contraction profiles in IABs.

### 6.3 Major Correlations in Our Study

We identified key factors influencing global LA strain, finding that PALS independently correlates with P-wave duration and HF. PALS indicates LA early diastole, suggesting that longer P-wave duration implies more impaired conduction and diastole of both LV and LA, making PALS a potential HF diagnostic variable. Parameters of IABs, P-wave measurements, LA strain, and HF correlate, providing a foundation for further research on HFpEF pathomechanisms.

**Fig. 5.**
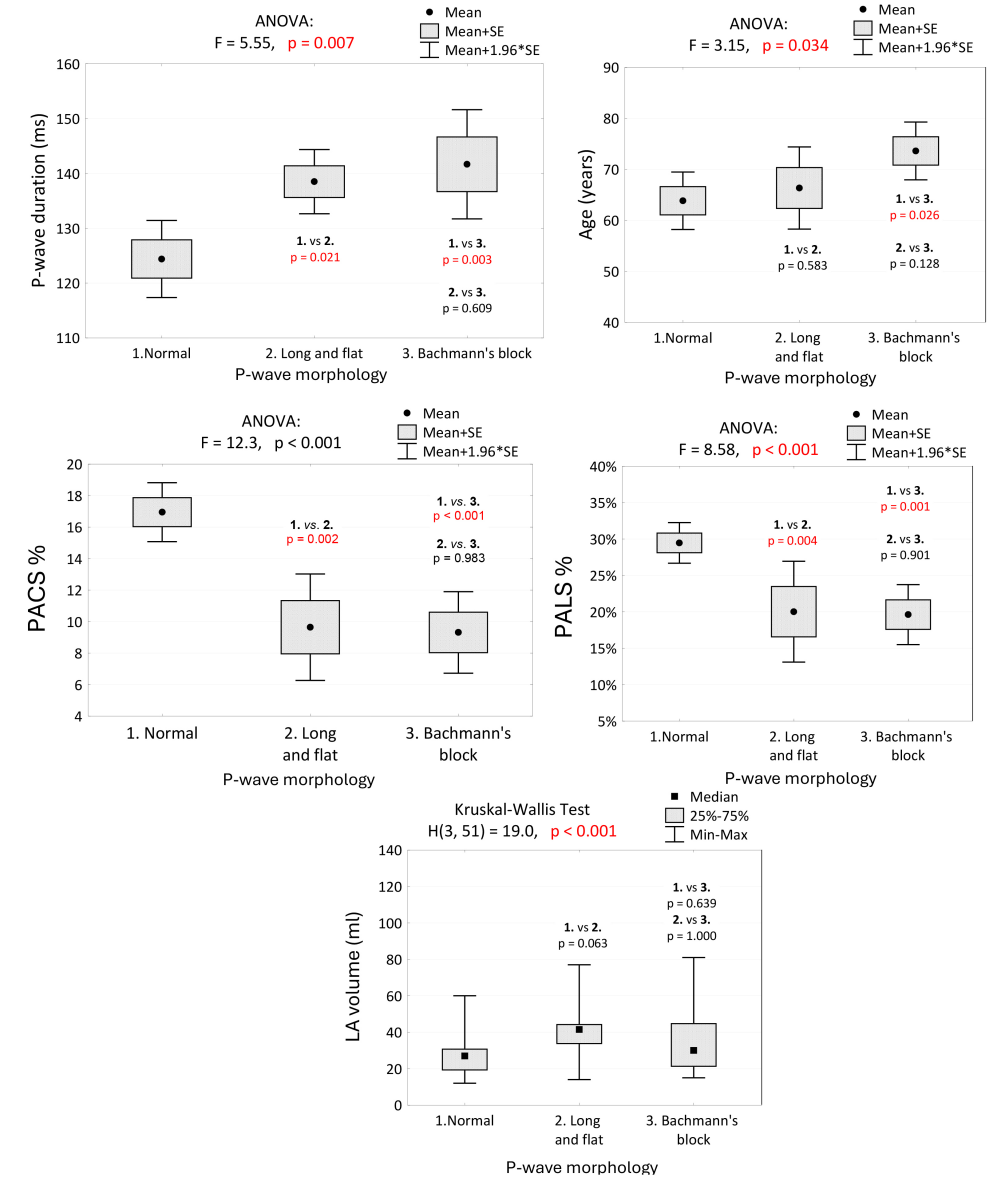
**P-wave duration, age, PALS, PACS and LA volume in groups of patients with regard to P-wave morphology, and the results of tests of independence and multiple comparisons (post-hoc tests)**. We noted a significant difference in P-wave duration between normal conditions and P-IAB and A-IAB. No significant difference was observed between P-IAB and A-IAB. Differences in PALS and PACS were also statistically significant only between normal and abnormal P-wave morphologies. No statistical differences were present between P-IAB and A-IAB. PALS, peak atrial longitudinal strain; PACS, peak atrial contraction strain; LVEF, left ventricular ejection fraction; PV, pulmonary veins; LA volume, left atrial volume; SE, standard error; ANOVA, analysis of variance; P-IAB, partial interatrial block; A-IAB, advanced interatrial block.

Discussing HF and impaired atrial conduction, we must mention “Bayes’ Syndrome” [31, 32]. Bayes de Luna concluded that in HF patients, A-IAB predicts new-onset atrial fibrillation (AF) and ischemic stroke. Our study found strong correlations between:

(1) The presence of IABs and episodes of PAF (Table 3, *p* < 0.001).

(2) A-IAB and decreased PALS and PACS (Table 2, *p* < 0.001; Fig. 5, 1 vs. 2 *p* < 0.004, 1 vs. 3 *p* < 0.001).

(3) Decreased PALS, PACS, and HF (reduced LVEF). (Fig. 2, *p* < 0.003).

**Table 3.** **Number (percentage) of patients in groups differing in the P-wave morphology and the results of tests of independence**.

Comorbidities	P-wave morphology			*p*-value
	Normal	Long and flat	Bachmann’s block	
	N = 18	N = 12	N = 15	
Arterial hypertension, *n* (%)	10 (55.6)	8 (66.7)	15 (100.0)	0.023
CKD, *n* (%)	1 (5.6)	0 (0.0)	1 (6.7)	0.762
HF, *n* (%)	2 (11.1)	3 (25.0)	4 (26.7)	0.264
IHD, *n* (%)	3 (16.7)	3 (25.0)	5 (33.3)	0.695
Asthma, *n* (%)	0 (0.0)	1 (8.3)	1 (6.7)	0.469
COPD, *n* (%)	3 (16.7)	1 (8.3)	2 (13.3)	0.925
Obesity, *n* (%)	0 (0.0)	0 (0.0)	4 (26.7)	0.038
DM2, *n* (%)	7 (38.9)	2 (16.7)	8 (53.3)	0.136
AFP, *n* (%)	0 (0.0)	2 (16.7)	4 (26.7)	<0.001

CKD, chronic kidney disease; IHD, ischemic heart disease; COPD, chronic obstructive pulmonary disease; DM2, diabetes mellitus type 2; AFP, paroxysmal atrial fibrillation; HF, heart failure.

Interestingly, we found no clear correlation between retrograde PV flow and IAB type. The topic seems to be scarcely explored in the literature. Reversed PV flow during atrial contraction, often discussed with regards to MR, did not correlate in our research [33, 34]. The complexity of IABs, as well as the potential subtypes warrant further study.

### 6.4 Correlations with Comorbidities

Our study identified correlations between arterial hypertension, being overweight, and IAB prevalence. Guozhe Sun *et al*.’s study [35] of 11,956 patients found a higher IAB prevalence in hypertensive individuals, consistent with our findings. IAB prevalence also increased with body mass index (BMI). Comorbidities such as DM2, chronic kidney disease, ischemic heart disease, asthma, and COPD did not correlate significantly with LA strain in our study. Vyas V. and Lambiase P’s epidemiological analysis [36] showed connections between obesity, DM2, and AF risk. Obesity was the primary factor in a large cohort study of 67,238 patients [37]. Long-term hyperglycemia in DM2 leads to myocardium fibrosis, increasing AF susceptibility [38]. These factors may independently influence atrial strain, but the exact mechanism is unclear, warranting further research. Pavasini *et al*.’s study [39] discussed the negative impact of COPD and stable coronary artery disease (CAD) on LA strain. However, the independence of CAD in this context was not apparent. Our study group (COVID-19) presents potential respiratory complications despite not needing mechanical ventilation, questioning the objectivity of COPD or asthma as independent factors.

## 7. Study Limitations

The primary limitation of our study was the small sample size, which restricted our ability to make precise comparisons among groups based on interatrial conduction. Another limitation is the lack of extensive clinical research on this topic, making it challenging to discuss our findings without sufficient comparative studies. However, what is worth noticing is that in 2022, Bucciarelli-Ducci *et al*. [40] published a review of the most relevant literature on the role of cardiovascular imaging in cardiovascular medicine about COVID-19. It emphasized that despite the limited access to hospital-based cardiovascular care, the developing imaging technology and artificial intelligence facilitated the understanding of myocardial damage caused by coronavirus. The authors presented examples connected with valvular diseases, coronary diseases, and hypertension. However, there needs to be sufficient information about the effects of COVID-19 on the cardiac conduction system, which stays within the scope of our interest.

We agree that acute COVID-19 is a significant limitation, which could have influenced the results. On the other hand, the specific group of patients provides unique data that can be compared with other groups in future studies. In his letter to the Editor, Russo Vincenzo referred to Yenercağ’s work, emphasizing that IABs may be revealed or amplified by COVID-19, which can potentially worsen the patient’s condition [41, 42]. We selected COVID-19-positive patients with this consideration in mind.

COVID-19 infection often acts as both a catalyst and a cause for IAB development. We highlight the need for further studies to establish average reference values for LA strain in normal P-waves. Pathan’s meta-analysis (2017) showed that average LA strain values in healthy P-waves differ slightly from those in our COVID-19 patients [43]. The reservoir phase values were 39% compared to our 29.97%, with no significant differences in the contraction phase (17% vs. 17.63%).

On the other hand, Nyberg *et al*. [44] 2023 published original research that included 1329 healthy patients. The research aimed to “establish echocardiographic reference ranges, including lower normal limits of global strains for all four cardiac chambers”. The LA reservoir strain (4ch) was, on average, 32% compared to our 29.97%. The LA contractile strain (4ch) was 15.4% vs. 17.63% respectively. However, we should pay attention to the age of the groups included in the research regarding LA strain. The mean age in our research was 69 years, and for that age, Nyberg assessed the LA reservoir strain (4ch) of 30.1% (females) and 30.6% (males), nearly equaling the 29.97% found in our study. Furthermore, the LA contraction strain was 15.8% (females) and 17.9% (males) vs. 17.63%, respectively, presenting no significant difference in our study. These comparisons attract our attention and make us ask further questions about the influence of COVID-19 on cardiac health.

## 8. Clinical Implications

The impaired function of the LA muscle associated with IABs presents a pathophysiological target for treating patients with HFpEF. Precise P-wave assessment is crucial in determining the condition of the LA.

## 9. Conclusions

(1) IABs negatively impact the mechanical profile of LA contraction.

(2) PALS (%) is correlated with P-wave duration (ms), LVEF (%), LA volume (ml), and the presence of MR.

(3) PACS (%) is correlated with LVEF (%), LA volume (ml), and P-wave morphology.

(4) Examining LA mechanics using regional strain complements precise electrocardiographic assessment.

(5) The influence of IABs on retrograde flow in PV is unclear, possibly due to insufficient patients with specific conduction disorders.

## Availability of Data and Materials

The datasets used and/or analyzed during the current study are available from the corresponding author on reasonable request.
